# Prevalence of metabolic syndrome among patients with schizophrenia in Ethiopia

**DOI:** 10.1186/s12888-021-03631-2

**Published:** 2021-12-11

**Authors:** Feyissa Challa, Tigist Getahun, Meron Sileshi, Zeleke Geto, Teshome S. Kelkile, Sintayehu Gurmessa, Girmay Medhin, Miraf Mesfin, Melkam Alemayehu, Tigist Shumet, Anwar Mulugeta, Desalegn Bekele, Christina P. C. Borba, Claire E. Oppenheim, David C. Henderson, Abebaw Fekadu, Anna Carobene, Solomon Teferra

**Affiliations:** 1 National References Laboratory for Clinical Chemistry, Ethiopian Public Health Institute, Gulelle Arbegnoch Street (the former Pasteur Institute): Gulele Sub City, Addis Ababa, Ethiopia; 2 Department of Biomedical Science, College of Medicine and Health Science, Wollo University, Dessie, Wollo Ethiopia; 3 Horizon Health Network, Fredericton, NB Canada; 4 Department of Psychiatry, School of Medicine, College of Health Sciences, Addis Ababa University, Addis Ababa, Ethiopia; 5 Aklilu Lemma Institute of Pathobiology Addis Ababa University, Addis Ababa, Ethiopia; 6 Australian Centre for Precision Health, University of South Australia, Adelaide, Australia; 7 Boston University School of Medicine, Boston, MA USA; 8 Boston Medical Center, Boston, MA USA; 9 Centre for Innovative Drug Development and Therapeutic Trials for Africa (CDT-Africa), College of Health Sciences, Addis Ababa University, Addis Ababa, Ethiopia; 10 Global Health & Infection Department, Brighton and Sussex Medical School, Brighton, UK; 11 King’s College London, Centre for Affective Disorders, Department of Psychological Medicine, Institute of Psychiatry, Psychology and Neuroscience, London, UK; 12 Laboratory Medicine, IRCCS San Raffaele Scientific Institute, Milan, Italy

**Keywords:** Metabolic syndrome, Prevalence, Schizophrenia, Ethiopia

## Abstract

**Background:**

Globally, the prevalence of metabolic syndrome (MetS) is higher among patients with schizophrenia than the general population, and this leads to higher morbidity and mortality in this population. The aim of this study was to investigate the MetS prevalence among patients with schizophrenia in Ethiopia.

**Methods:**

We conducted a cross-sectional analysis of baseline data of 200 patients with schizophrenia recruited from Amanuel Mental Specialized Hospital, Addis Ababa, Ethiopia. Lipid profile and blood glucose levels were measured using Roche Cobas 6000 clinical chemistry analyzer. The prevalence of MetS was assessed based on National Cholesterol Education Program Adult Treatment Panel III criteria. Patients’ demographic information, clinical and laboratory data, lifestyle habits, particularly smoking and Khat chewing, were evaluated vis-à-vis MetS.

**Results:**

The overall prevalence of MetS in patients with schizophrenia was 21.5% (17.1% male, 29.6% female) where Low HDL-cholesterol value was the most common metabolic disorders components in both males and females subgroups. In the multivariate analysis, the positive and negative symptoms score (PANSS, AOR = 1.03, 95% CI 1.001–1.054) was associated factors with MetS.

**Conclusion:**

In Ethiopia, patients with schizophrenia were found to have higher prevalence of MetS than the general population. Physicians/health care providers should routinely screen patients with schizophrenia for MetS and initiate timely management of those who develop the syndrome to reduce the health cost from caring for NCDs, improve the patients’ quality of life, and prevent premature mortality.

## Introduction

Schizophrenia is one of severe mental disorder that affects 1% of the total population globally and is accompanied by serious functional impairments [1]. Patients with schizophrenia have approximately 20% reduced life expectancy compared to the general population [2]. Schizophrenia is in fact a life-threatening disease associated with mortality rates that are two to three times higher than those expected/observed in the general population [3], and in a study in Ethiopia based on a 5 years follow up, up to six fold increase in overall mortality has been reported [4]. Suicide is one of the factors for death in patients with schizophrenia [5], but the high mortality is also related to natural causes such as respiratory diseases [6], cancer [7], and cardiovascular diseases (CVDs) [8]. The high prevalence of unhealthy dietary habits, sedentary lifestyle, obesity, and smoking habits among patients suffering from schizophrenia have been referred as significant contributing factors for a higher than normal risk of developing CVDs, constellation of clinical findings that identify the metabolic syndrome [9].

Metabolic syndrome (MetS), known also as” *Insulin Resistance Syndrome*” or “*Syndrome X*”, comprises several clinical aspects attributable to a higher than normal risk of developing diabetes mellitus or CVDs which is characterized by high fasting blood glucose and high triglycerides concentrations, low level of high density lipoprotein (HDL), and high waist circumference [10].

Several criteria are used to define the MetS, like the definition from the National cholesterol Education Program Adult Treatment Panel (commonly referred as ATP III or NCEP criteria), the Adopted definition (NCEP ATP III A), from the World Health Organization (WHO), from the American Diabetes Association (ADA’s), and also from the Japan Society for the study of Obesity (JASSO) [11]. The two definitions most commonly used are the ATP III A criteria, proposed by American Heart Associations (AHA), and the WHO definition, that, differently from ATP III, includes also the albuminuria and abnormal glucose regulation [12].

Globally, the prevalence of MetS among patients with schizophrenia is twice higher than the general population [13]. In Ethiopia, the prevalence of MetS among the general population has been recently determined in a large sample size, but evidence about the prevalence of MetS among patients with schizophrenia in low income countries is scarce. Few studies were conducted in Ethiopia, particularly in capital city (Addis Ababa), southern and western part of Ethiopia, to assess the prevalence of MetS among patients with psychiatric illness [14–16]. The aim of this study was to investigate the prevalence of MetS among patients with schizophrenia in Ethiopia. The findings from this study will inform clinicians in Ethiopia to institute appropriate interventions to prevent the development of MetS. It will also add to the body of knowledge on MetS among schizophrenia patients from LMICs.

## Methods

### Study participants

We conducted a cross-sectional analysis of a baseline data of two hundred outpatients study participants recruited at the Amanuel Mental Specialized Hospital, Addis Ababa, Ethiopia from January, 2015 and November, 2016. Diagnoses were made by psychiatrists according to Diagnostic and Statistical Manual of Mental Disorders Fourth edition (DSM-IV) [17] as part of folate clinical trial, (Clinicaltrials.gov identifier: NCT01724476), a 4 month trial of Folate plus Vitamin B12 in Ethiopia) [18]. Patients who had positive and negative symptoms score (PANSS) of 60 or higher were included in the study. The study was reviewed and approved by both Addis Ababa University, College of Health Science, Institutional Review Board (Protocol #040/12/SPH) and Massachusetts General Hospital Review Board (Protocol #2011-P-002667). All study participants provided written informed consent.

### Assessment

At the baseline visit general information were collected from outpatients with schizophrenia by filling in an enrollment questionnaire by trained psychiatric nurse under four main headings:

1. Patient’s demographic information: age, sex, ethnicity, marital status, and occupational status.
2. Patient’s clinical data: height, weight, waist circumference (measured) and body mass index (BMI). Blood pressure was measured twice by standard mercury sphygmomanometers on the right hand after the study participants seated for 5 min and the average of the two readings was used for the data analysis. Both pharmacological treatment and illness duration data were collected from medical history.
3. Patient’s lifestyle habits: smoking and Khat chewing status was assessed (Yes/No response). Khat is an evergreen shrub (*Catha Edulis* Forsk) which has amphetamine like stimulant property and a common habit in East Africa [19].
4. Patient’s clinical laboratory data: total cholesterol, HDL- cholesterol, triglycerides, and fasting blood glucose concentrations were measured, as described below.

### Laboratory analysis

Whole blood (4-5 ml) was collected from study participants by experienced laboratory technologist after overnight fasting for 8 h and allowed to clot for 30 min before centrifugation at 4500 rpm for 5 min. Serum samples were separated and total cholesterol, HDL cholesterol, triglycerides, and glucose were measured the same day using Roche Cobas 6000 analyzer (Roche Diagnostics GmbH, Mannheim, Germany). The measurements were performed at the National Reference Laboratory for Clinical Chemistry of Ethiopia Public Health Institute (EPHI). This laboratory undergoes an external quality assessment scheme and is accredited by the Ethiopia National Accreditation Office (ENAO). Diagnosis of MetS was established using the NCEP/ATPIII A criteria (Table 1).

**Table 1:** Criteria for metabolic syndrome classification according to the NCEP/ATPIII A

	NCEP ATP III A
Definition	Any three of the following 5 features
Waist Circumference (cm)	Male ≥102, Female ≥88
Blood Pressure (mm Hg)	Systolic ≥130, or Diastolic ≥85
Triglyceride (mg/dl)	≥ 150
Fasting blood glucose (mg/dl)	≥100
HDL Cholesterol (mg/dl)	M < 40, F < 50

Abbreviations: *NCEP ATP III A* National Cholesterol Education Programs Adult Treatment Panel III

### Statistical analysis

The descriptive statistics used for the categorical data were frequencies and percentages, while for the continuous data mean, median, standard deviations (SD), and interquartile range (IQR) were applied. Differences between categorical and continuous data were evaluated using Pearson Chi-square test or Fisher’s exact test and logistic regression, respectively. In multivariate analysis model, after removing parameters used in the diagnosis of MetS, variables with *p*-value lower than 0.05 were included in the analysis. The presence of MetS was used as dependent categorical variable in both univariate analysis and multiple logistic regressions. A *p*-value < 0.05 was considered significant. Statistical analysis was performed using SPSS Version 22.00 (SPSS Inc. Chicago, IL, USA). The figures were performed using python 3.7.

## Results

### Demographic and clinical characteristics

Socio-demographic characteristics of participants stratified according to the gender are presented in Table 2. A total of 200 (129 males, 71 females) patients with schizophrenia were recruited in this study, with mean age 38.4 ± 9.8 years. One-third of patients with schizophrenia had high BMI (≥25 Kg/m^2^). The mean duration of the illness in the patients was 14 ± 9 years, and 66% of the patients were using the first-generation antipsychotic (FGA) medication while only 34% the second generation of antipsychotic (SGA). Approximately 21% were smokers, 8% gave a history of chewing Khat, 78% were not working, 86% were single during the study period, and over two-thirds lived with parental family.

**Table 2:** Demographic and clinical characteristics of study subjects

	Total	Male	Female
Number of subjects, n (%)	200	129(64.5)	71(35.5)
Age (in years) mean ± SD	38.4 ± 9.8	38.4 ± 10.1	38.4 ± 9.4
Waist circumference in cm mean ± SD	89.3 ± 9.7	88.4 ± 8.7	91 ± 11
BMI (Kg/m^2^) ≥ 25 n (%)	73(36.5)	32(24.8)	41(57.7)
Ethnicity n (%)			
Amhara	82(41.0)	48(37.2)	34(47.9)
Oromo	45(22.5)	30(23.3)	15(21.1)
Tigray	8(4.0)	5(3.9)	3(4.2)
Gurage	42(21.0)	28(21.7)	14(19.7)
others	23(11.5)	18(14.0)	5(7.0)
Systolic Blood pressure (mm Hg) mean ± SD	120 ± 18	121 ± 17	118 ± 21
Diastolic Blood Pressure (mm Hg) mean ± SD	78.4 ± 11.5	78.7 ± 11.7	78.0 ± 11.1
Pharmacological treatment n (%)			
FGAs	132(66.3)	88(68.8)	44(62.0)
SGAs	67(33.7)	40(31.2)	27(38.0)
Lives with parental family n (%)	160(80.0)	112(86.8)	48(67.6)
PANSS Score mean ± SD	83.6 ± 15.4	84.0 ± 15.4	82.9 ± 15.6
Smokers n (%)	42(21.4)	41(32.5)	1(1.4)
Khat chewing Status n (%)			
Yes	16(8.2)	16(12.7)	0(0)
No	180(91.8)	110(87.3)	70(100)
Occupational Status, working n (%)	44(22.0)	29(22.5)	15(21.1)
Duration of illness (Years), mean ± SD	14 ± 9	15 ± 9	14 ± 9
Marital Status, Married n (%)	28(14.0)	15(11.6)	13(18.3)
Total Cholesterol (mg/dl), median (IQR)	183(216–157)	176(211–154)	198(221–174)
Triglycerides (mg/dl), median (IQR)	118(164–83)	123(179–87)	103(149–77)
HDL-Cholesterol (mg/dl), median (IQR)	45(54–38)	43(49–36)	50(60–44)
FBG(mg/dl), median (IQR)	91(100–86)	91(98–86)	91(103–86)

*Abbreviations: COR* Crude odds ratios, *SD* standard deviation, *BMI* Body mass index, *WC* Waist Circumference, *BP* blood pressure, *PANSS* Positive and Negative Syndrome Scale, *FGAs* first generation antipsychotics, *SGAs* second generation antipsychotics, *HDL* high-density lipoprotein, *FBG* Fasting blood glucose, *IQR* interquartile range

### Prevalence of metabolic syndrome and its components

**Fig. 1:**
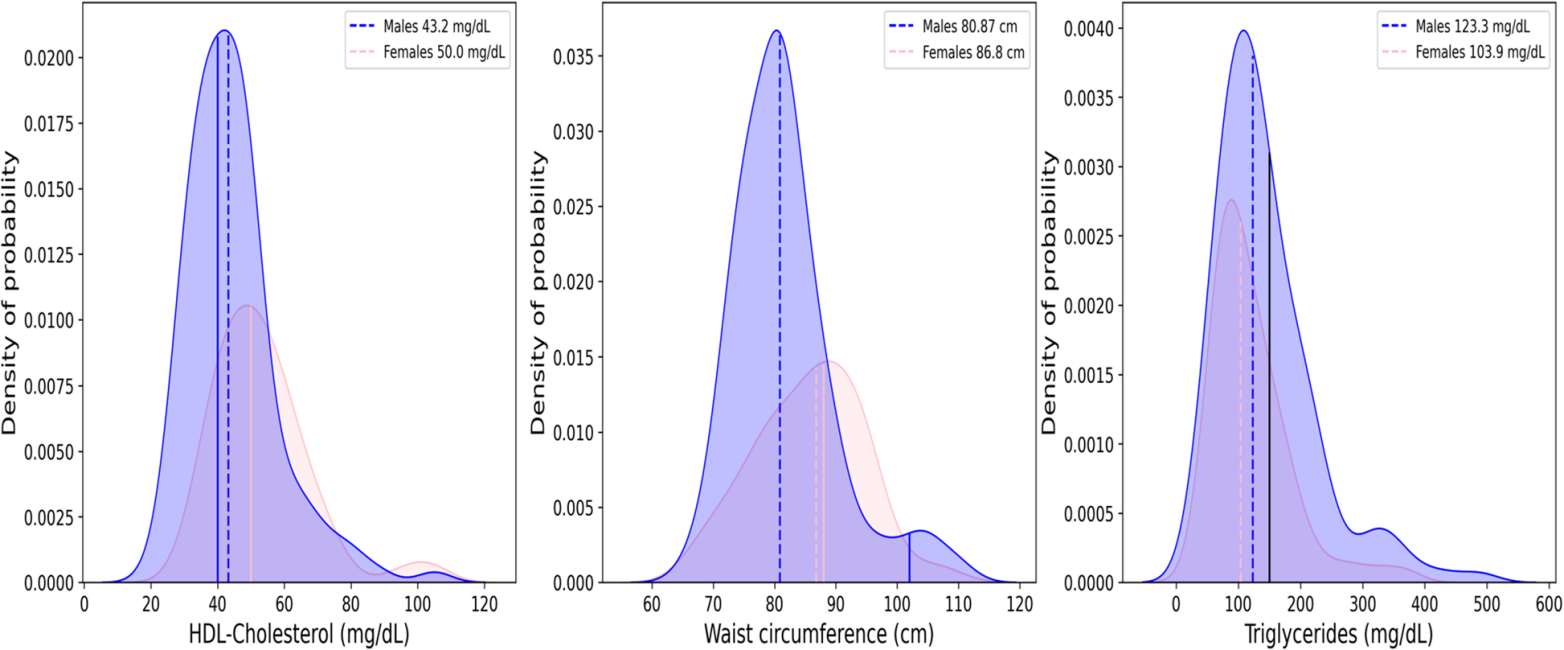
Distributions of HDL-Cholesterol (panel **A**), waist circumference (panel **B**), and triglycerides (panel **C**) values for males and females (blue and pink area, respectively). Dotted lines represent the median values and the continuous line the limits defined as criteria for the metabolic syndrome (MetS) classification differentiated for sex, if necessary, as reported in Table 1. HDL Cholesterol median value for female subgroup is overlapped to the criteria defined for the MetS (50 mg/dL)

Low HDL-cholesterol value was the most common metabolic disorders component in both males and females subgroup as shown in the Fig. 1. High waist circumference was present in the female group with a high percentage, but not common in the male subgroup, while high triglycerides level was the least metabolic disorder component in both females and males subgroup (Fig. 1, Table 3).

**Table 3:** Prevalence of metabolic abnormalities in patients with schizophrenia n (%)

NECP ATP III	Total	Male	Female	OR female to male (95% CI)	*P* value	
WC (M > 102 cm; F > 88 cm)	50(25.0)	9(7.0)	41(57.7)	18.2(8.0–41.6)	< 0.001	
Triglycerides (≥ 150 mg/dl)	61(30.7)	44(34.4)	17(23.9)	0.6(0.3–1.2)	0.126	
BP (≥ 130/85 mmHg)	74(37.0)	50(38.8)	24(33.8)	0.8(0.4–1.5)	0.487	
HDL Cholesterol (M < 40 mg/dl; F < 50 mg/dl)	88(44.4)	54(42.5)	34(47.9)	1.24(0.7–2.2)	0.466	
FPG (≥100 mg/dl)	49(24.9)	27(21.3)	22(31.4)	1.7(0.8–3.3)	0.114	

Abbreviations: *NCEP ATP III* National Cholesterol Education Programs Adult Treatment Panel III, *WC* Waist Circumference, *BP* blood pressure, *HDL* high-density lipoprotein, *M* males, *F* females, *FBG* Fasting blood glucose

The overall prevalence of MetS among patients with schizophrenia, according to NCEP ATP III A criteria was 43(21.5%) (22 males and 21 females), while, 157 patients (78.5%), presented 2 or less MetS criteria (Table 4).

**Table 4:** Prevalence of metabolic abnormalities according to the NCEP ATP III A criteria among patients with schizophrenia in Ethiopia

Number of MetS criteria	Total n (%)	Gender	
		Male n (%)	Female n (%)
0	45(22.5)	35(27.1)	10(14.1)
1	57(28.5)	36(27.9)	21(29.6)
2	55(27.5)	36(27.9)	19(26.8)
3	25(12.5))	16(12.4)	9(12.7)
4	12(6.0)	4(3.1)	8(11.3)
5	6(3.0)	2(1.6)	4(5.6)
MetS≥3 criteria	43(21.5)	22(17.1)	21(29.6)

Abbreviations: *MetS* metabolic syndrome

### Associated factors with metabolic syndrome

To determine the associated factors to the MetS, patients were divided in with 43(21.5%) and without MetS 157(78.5%) subgroups. Age, BMI, marital status (married/single), occupational status (working/not working), pharmacological treatment (FGAs/SGAs), Khat Chewing use (Yes/No), and living arrangement (with parental/marital family or other), were found as factors not associated to the MetS. Gender, PANSS score, and smoking habit were found associated with MetS in univariate analysis. In multivariate analysis only PANSS score were found associated factor with MetS in patients suffering from schizophrenia (Table 5).

**Table 5:** Univariate and multivariate analysis to evaluate associated factors to the metabolic syndrome in patients suffering from schizophrenia in Ethiopia (*n* = 200)

Variable	Metabolic Syndrome		COR(95%CI)	***P*** = value	AOR(95%CI)	***P*** = value
Number of patients (%)	Yes, 43(21.5))	No, 157(78.5)				
Age (in Years) n (%)						
< 30	3(7.0)	30(19.1)	1			
≥ 30	40(93.0)	127(80.9)	0.32(0.09–1.1)	0.70		
**Sex** n (%)						
Male	22(51.2)	107(68.2)	1		1	
Female	21(48.8)	50 (31.8)	**2.04(1.03–4.06)**	**0.041**	0.63(0.30–1.34)	0.231
**Marital status** n (%)						
Married	9(20.9)	19(12.1)	1			
Single	34(79.1)	138(87.9)	1.92(0.8–4.6)	0.144		
**Occupation status** n (%)						
Working	11(25.6)	33(21.0)	1			
Not working	32(74.4)	124(79.0)	1.29(0.59–2.83)	0.523		
**PANSS Score mean ± SD**	78 ± 13	85 ± 16	**1.03(1.01–1.06)**	**0.012**	**1.03(1.001–1.054**	**0.046**
**Duration of illness (in Years**) mean ± SD	16 ± 9	14 ± 9	0.98(0.94–1.02)	0.288		
**Current treatment** n (%)						
FGAs	29(67.4)	103(66.0)	1			
SGAs	14(32.6)	53(34.0)	0.94(0.46–1.93)	0.862		
**Smoking status** n (%)						
No	37(92.5)	117(75.0)	1			
Yes	3(7.5)	39(25.0)	**4.1(1.2–14.1)**	**0.024**	3.2(0.88–11.7	0.077
**Khat Chewing status** n (%)						
No	37(92.5)	143(91.7)	1			
Yes	3(7.5)	13(8.3)	1.12(0.30–4.14)	0.864		
**Living arrangement** n (%)						
Lives with parental family	30(69.8)	130(82.8)	1			
Lives with marital family	8(18.6)	17(10.8)	0.49(0.19–1.24)	0.133		
Lives with other relatives/lives alone	5(11.6)	10(6.4)	0.46(0.15–1.50)	0.185		

Abbreviations: *AOR* adjusted ratios, *COR* Crude odds ratios, *SD* standard deviation, *PANSS* Positive and Negative Syndrome Scale

**Fig. 2:**
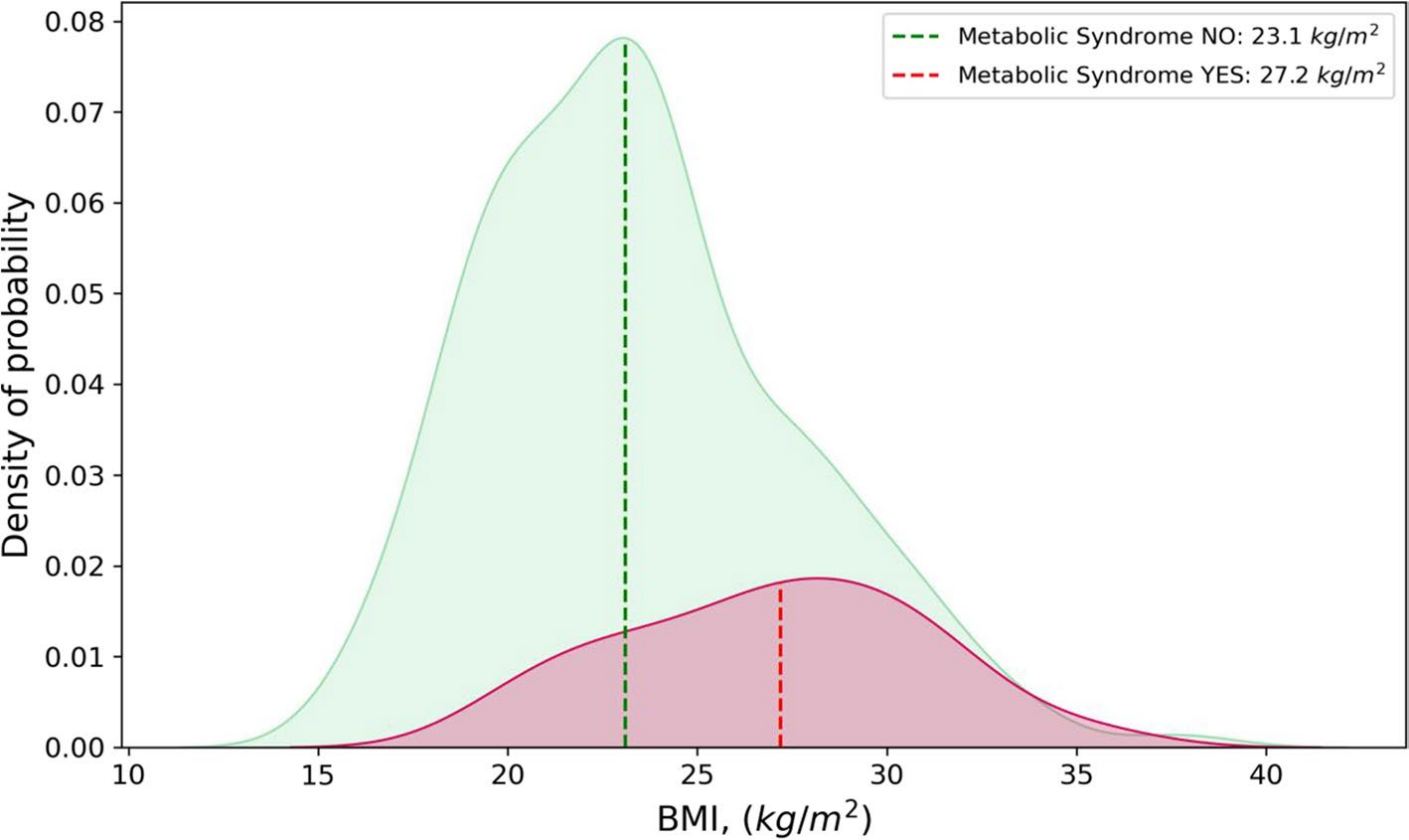
Distributions of Body Mass Index (BMI) values, for patients with and without diagnosis of metabolic syndrome (MetS) (red and green area respectively). Dotted lines represent the median values of the subgroup-

**Fig. 3:**
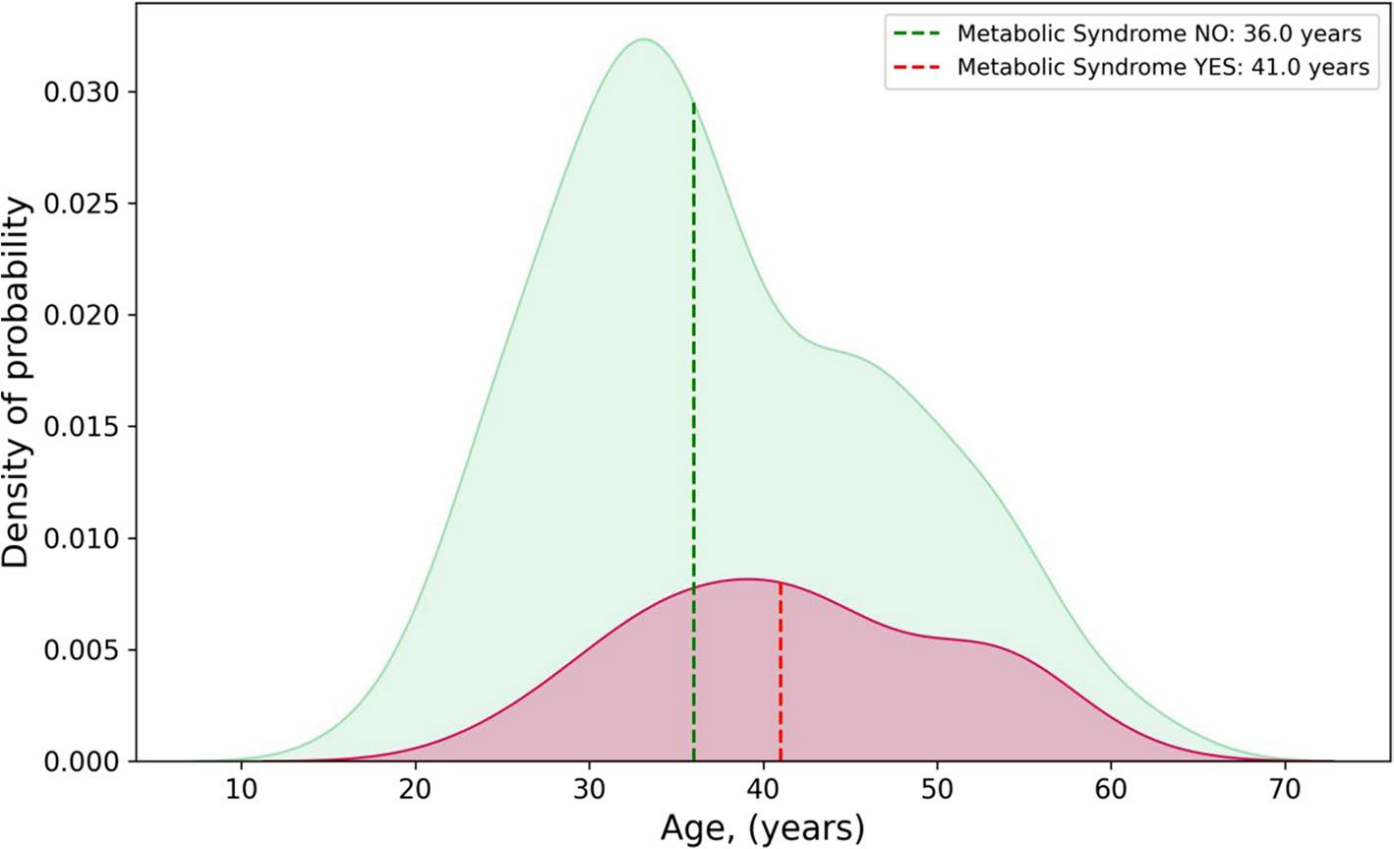
Distributions of ages, for patients with and without diagnosis of metabolic syndrome (MetS) (red and green area respectively). Dotted lines represent the median values of the subgroup

In Figs. 2 and 3 are shown ages and BMIs distributions among people with and without MetS.

## Discussion

In this study we reported the prevalence and associated factors for metabolic syndrome among patients with schizophrenia which is the first study to compare the findings to the national population prevalence of MetS. The Ethiopian community based survey, conducted from April to June 2015, in a population of 10,260 adults people, quoted a prevalence of metabolic syndrome of 4.8% (8.6% in females and 1.8% in males respectively) [20]. In the current study, we investigated the prevalence of MetS among two hundreds patients suffering from schizophrenia in Ethiopia. The overall prevalence of MetS among these patients was 22% according to the ATP III A criteria with higher proportion among females compared to males (29.6 and 17.1% respectively).

The etiology of the MetS among patients with schizophrenia is multifactorial and this includes psychotropic drugs such as second-generation antipsychotics, immune-metabolic dysregulations, and lifestyle risk factors (e.g. physical inactivity, smoking, excessive alcohol intake, poor sleep, and unhealthy nutritional patterns) [9]. The immune dysregulations include increased levels of inflammatory markers such as C - reactive protein (CRP), interlukin-6 (IL-6), tumour necrosis factor alpha (TNF alpha), and other cytokines indicating insulin and leptin resistance, obesity, inflammation, and higher rates of metabolic syndrome [21]. Recent study indicated that plasma apelin level higher among schizophrenia patients which may be related to severity of mental illness which impact on MetS [22].

A recent systematic review and meta-analysis by Vancampfort and et al. [23] indicated that the pooled prevalence of MetS among patients with severe mental illness ranged between 25 and 50%, and the finding of our current study is comparable with the range reported globally. Other evidences among patients with schizophrenia indicated prevalence of MetS of 42% (51.6% of women, 36% men) in a study conducted in USA [24], a prevalence of 42.6 and 48.5% for men of women respectively in a Canadian study [25], 27.5% in Japan [26], 43.6% in Palestine [27], 46.7% in Malaysia [28], 40% in Turkey [29], and 40% in India [30].

Studies conducted in different part of the African continent indicated that the prevalence of MetS is comparable with the global data. For instance, in a study carried out in South Africa on 278 subjects with severe mental illness, the estimated prevalence of MetS was 23.2% [31]; whereas, in the middle belt of Ghana 14.1% [32]. The report from Kenya was slightly higher at 28.6% [33]. Three studies conducted in the central, south, and western part of the Ethiopia among patients with psychiatric illness, diagnosed with schizophrenia, major depressive disorder, bipolar disorder and others, reported prevalence of MetS ranged from 18 to 25% [14–16]. Our finding is comparable to those reported by previous studies even though our study include only patients diagnosed with schizophrenia.

Similar to our study, higher prevalence of MetS was reported from different studies among females compared to male patients with schizophrenia [24, 25], and this was consistent with the MetS finding from the Ethiopian general population survey [20]. One important reason for high MetS among females might be due to use of hormonal contraceptives [34].

The present study found increasing PANSS score associated with MetS. The relationship between increasing PANSS score and MetS has been already reported in patients with schizophrenia [35, 36] and this could be explained by certain habits such as sedentary lifestyle and /or dietary habits as factors contributing to obesity.

There is inconsistent reports on the relation between the different generations of antipsychotic medications and MetS among patients with schizophrenia [36–39]. In this study, we did not find significant difference in the development of MetS between first generation and second generation antipsychotic medications (FGAs or SGAs). Even though there is inconsistent report between MetS and antipsychotic medication, increasing evidence indicates that the disease itself is an independent risk factor to develop MetS, as demonstrated by the presence of higher rates of MetS in antipsychotic naïve patients [40].

The current approach for the diagnostic criteria for MetS, the cut-off point of waist circumference has been debated. Most published literature widely used criterion in ATPIII defined 102 cm in men and 88 cm in women as the cut-off points to diagnose MetS, mainly developed based on data from western population. In addition the IDF criterion (2005) suggest the cut-off values of waist circumferences to be 90 cm in men and 80 cm in women [12]. In our study, as reported in Table 3, only 9 males (7%) presented a value of waist circumference higher than the cut off, while the prevalence of waist circumference abnormality in females is > 50% (Table 3, Fig. 1). A cross-sectional community-based study in nine Ethiopian regions, including more than 5.000 recruited participants, reported a percentage of obesity among the population of 2.2% (BMI > 30) [41] while in US the obesity is a common disease regarding more than 40% of the population [42]. Africa which have great cultural, linguistic, and historic diversity the cut-off point of waist circumferences depends on western population which might not to be appropriate for Africa population, at least for Ethiopia.

Even though the prevalence of metabolic syndrome among patients suffering from schizophrenia is high, the rate of screening for this syndrome among these patients is still low, especially in low and middle-income countries [43]. A recent meta-analysis of prospective cohort studies by Wu et al. [44], showed that individuals with MetS had had 46% increased risk of mortality when compared with individuals without MetS. The higher prevalence of metabolic syndrome among patients with schizophrenia compared with the general population has several clinical consequences for this vulnerable group such as the development of chronic diseases and subsequent premature mortality. The consequence is grave in LIMICs where quality health services are low or inaccessible.

### Limitations

The current study has some limitations. First, we conducted a cross-sectional analysis of baseline data study which referred only about a single point in study. Second, we applied only one criterion (ATP III A criteria) to estimate the prevalence of MetS among patients with schizophrenia and not use control to make comparison. Third, potential factors for the development of MetS such as genetic variations, and lifestyle such as physical exercise and diet were not included.

## Conclusion

The prevalence of Mets among patients with schizophrenia in Ethiopia is much higher than the general population. The current finding highlights the need for screening of MetS components in patients with schizophrenia, particularly those who have high BMI and older patients.

## Data Availability

The datasets used and analyzed during the current study are available from the first Corresponding author Feyissa Challa on reasonable request.
